# The Emerging Role of Non-Coding RNAs in Osteoarthritis

**DOI:** 10.3389/fimmu.2021.773171

**Published:** 2021-11-29

**Authors:** Soudeh Ghafouri-Fard, Christophe Poulet, Michel Malaise, Atefe Abak, Bashdar Mahmud Hussen, Afshin Taheriazam, Mohammad Taheri, Mohammad Hallajnejad

**Affiliations:** ^1^ Department of Medical Genetics, School of Medicine, Shahid Beheshti University of Medical Sciences, Tehran, Iran; ^2^ Department of Rheumatology, University Hospital of Liège (CHULiege), Liège, Belgium; ^3^ Fibropôle Research Group, University Hospital of Liège (CHULiege), Liège, Belgium; ^4^ GIGA-I3 Research Group, GIGA Institute, University of Liège (ULiege) and University Hospital of Liège (CHULiege), Liège, Belgium; ^5^ Men’s Health and Reproductive Health Research Center, Shahid Beheshti University of Medical Sciences, Tehran, Iran; ^6^ Department of Pharmacognosy, College of Pharmacy, Hawler Medical University, Erbil, Iraq; ^7^ Center of Research and Strategic Studies, Lebanese French University, Erbil, Iraq; ^8^ Department of Orthopedics, Tehran Medical Sciences Branch, Islamic Azad University, Tehran, Iran; ^9^ Institute of Human Genetics, Jena University Hospital, Jena, Germany; ^10^ Skull Base Research Center, Loghman Hakim Hospital, Shahid Beheshti University of Medical Sciences, Tehran, Iran

**Keywords:** lncRNA, miRNA, osteoarthritis, ncRNAs, expression, circRNA

## Abstract

Osteoarthritis (OS) is the most frequent degenerative condition in the joints, disabling many adults. Several abnormalities in the articular cartilage, subchondral bone, synovial tissue, and meniscus have been detected in the course of OA. Destruction of articular cartilage, the formation of osteophytes, subchondral sclerosis, and hyperplasia of synovial tissue are hallmarks of OA. More recently, several investigations have underscored the regulatory roles of non-coding RNAs (ncRNAs) in OA development. Different classes of non-coding RNAs, including long ncRNAs (lncRNAs), microRNAs (miRNAs), and circular RNAs (circRNAs), have been reported to affect the development of OA. The expression level of these transcripts has also been used as diagnostic tools in OA. In the present article, we aimed at reporting the role of these transcripts in this process. We need to give a specific angle on the pathology to provide meaningful thoughts on it.

## Introduction

As the most frequent degenerative condition in the joints, osteoarthritis (OA) has been associated with adults’ pain and disability. Joint damage, overweight, aging, and heredity factors are regarded as an etiologic factor for OA (1). Several abnormalities in the articular cartilage, subchondral bone, synovial tissue, and meniscus have been detected in the course of OA. Destruction of articular cartilage, the formation of osteophytes, subchondral sclerosis, and hyperplasia of synovial tissue are hallmarks of OA (1). Several molecules and pathways such as TGF-β, Wnt3a, Hedgehog, Smad3, β-catenin, and HIF-2α have been identified to contribute to the pathologic event during the OA course (1). In addition, systemic inflammation and the secreted cytokines in this process, particularly IL-1β and TNF-α can activate the NF-κB pathway in synovial cells and chondrocytes, participating in the pathogenesis of OA (1, 2). More recently, several investigations have underscored the regulatory roles of non-coding RNAs (ncRNAs) in OA development. Different classes of non-coding RNAs, including long ncRNAs (lncRNAs), microRNAs (miRNAs), and circular RNAs (circRNAs), have been reported to affect the development of OA. In the present article, we aimed at reporting the role of these transcripts in this process.

## LncRNAs in Osteoarthritis

LncRNAs are transcripts with sizes of more than 200 nucleotides. Although they are not translated into functional polypeptides, they have crucial functions in regulating protein-coding genes’ expression. As a novel epigenetic control level, they affect several human disorders’ pathogenic course (3). Despite poor evolutionary conservation across different species (4) and a low level of expression in many tissues (5, 6), their functionality in the regulation of gene expression in cis- and trans- modes has been verified (3). These transcripts can serve as molecular sponges for miRNAs to release miRNA targets from inhibitory effects of miRNAs. Moreover, they can induce the H3K27 trimethylation, as a repressive epigenetic mark in the promoter of certain genes, thus inactivating target genes (7).

In the course of OA, several lncRNAs have been reported to be dysregulated. Function of some of lncRNAs in the pathogenesis of osteoarthritis has been described with more details in research papers. In this section, we selected some of them with clearer mechanistical information. For instance, expression of H19 has been reported to be up-regulated in samples from OA patients and chondrocytes cultured in the presence of IL-1β (8). H19 up-regulation has suppressed proliferation and stimulated apoptosis in these chondrocytes, whereas H19 silencing has exerted the opposite impact. These effects are mediated through sponging miR-106a-5p (8). Notably, expression of this lncRNA has also been shown to be elevated in peripheral blood of patients with OA in correlation with the Kellgren and Lawrence (K-L) grading system. Besides, its expression has been inversely correlated with bone metabolism parameters, namely PINP, N-MID, BGP, BALP, and Lysholm score, while being positively correlated with β-CTX parameter and VAS and WOMAC scores (9). In addition to H19, expression of HOTAIR has been up-regulated in cartilage samples of the femoral condyles or tibial plateaus of patients affected with OA compared with control samples. Up-regulation of this lncRNA has led to a severe upsurge of apoptotic rate and decreased chondrocyte viability. Mechanistically, HOTAIR increases Bax expression and the proteolytic cleavage of caspase 3 and decreases survivin and Bcl-2 levels. In chondrocytes, functional studies have shown that HOTAIR acts as a sponge for miR-130a-3p, i.e. sequesters this miRNA and releases its targets from inhibitory effects of this miRNA (10). Another functional route for the participation of HOTAIR in the development of OA is through enhancement of expression of genes related to cartilage destruction. HOTAIR directly represses the expression of Wnt inhibitory factor 1 (WIF-1) through induction of H3K27 trimethylation in its promoter, thus activating the Wnt/β-catenin pathway (7). DANCR is another up-regulated lncRNA in human OA cartilage and lipopolysaccharide (LPS)-induced chondrocyte cells. DANCR silencing has attenuated LPS-associated apoptosis and inflammation, enhanced cell survival, abridged apoptosis, and reduced IL-1β, IL-6, IL-8, and TNF-α levels. DANCR functions are mediated *via* sponging miR-19a (11). In addition, DANCR has been recently found to regulate expression of SOX9 (12). DANCR has also been shown to enhance the proliferation of synovial fluid-derived mesenchymal stem cells and increase chondrogenesis through sponging miR-1275, a miRNA that regulates the expression of MMP13 and regulate its expression. Through similar route, DANCR regulates expression of SOX9 (13). Expression of MALAT1 has also been elevated in the synovial tissues of obese OA patients compared with normal-weight OA cases or non-OA controls. Its expression has been sharply activated following the induction of OA synovial fibroblasts with pro-inflammatory cytokines. MALAT1 silencing has reduced levels of CXCL8 in OA synovial fibroblasts while increasing TRIM6, IL7R, HIST1H1C, and MAML3 levels. Moreover, MALAT1 silencing has suppressed the proliferation of synovial fibroblasts of obese OA patients (14). NEAT1 and XIST are among other lncRNAs whose contribution to the pathogenesis of OA has been vastly investigated. Nevertheless, the results of the studies of their expression patterns are not consistent. In this section, we summarize studies that reported their up-regulation in OA. NEAT1 was described as a regulator of the OA development through sponging miR‐193a‐3p, miR-543, and miR-377-3p, thus increasing expressions of SOX5 and PLA2G4A. Subsequently, it affects chondrocyte proliferation and apoptosis and extracellular matrix (ECM) degradation (15–17). XIST increases OPN levels while decreasing miR-1277-5p, miR‐142-5p and TIMP-3 levels. Changes in these genes’ expression result in ECM destruction, induction of inflammatory responses, and abnormal proliferation/apoptosis of chondrocytes (18–21). **Table 1** shows the results of studies that demonstrated up-regulation of lncRNAs in OA tissues.

**Table 1:** Up-regulated lncRNAs in OA.

lncRNA	Clinical Samples	Assessed Cell Lines	Targets/Regulators	Signaling Pathways	Description	Reference
ARFRP1	83 OA and 29 normal tissues	–	miR-15a-5p, TLR4	NF-κB	Increased ARFRP1 levels result in elevated chondrocytes’ injury.	(22)
LOXL1-AS1	62 OA and 48 normal tissues	–	miR-423-5p, KDM5C/JUND1	–	LOXL1-AS1 improved the inflammation and proliferation rate in chondrocytes.	(23)
HOTAIR	Cartilaginous and normal control regions in 10 OA patients	–	miR-130a-3p, LC3-II/I, p62	–	By down-regulating miR-130a-3p levels, HOTAIR expands apoptosis and lowers autophagy and viability.	(10)
	10 OA and 10 control cartilage tissues	SW1353	WIF-1, β-Catenin, c-Myc, ZEB1, Snail	Wnt/β-catenin	HOTAIR improves trimethylation on histone H3K27 promoter region in WIF-1, resulting in WIF-1 down-regulation and Wnt pathway activation. Consequently, cartilage degrading genes were up-regulated.	(7)
H19	37 OA and 15 normal tissues	–	miR-106a-5p	–	H19 diminishes miR-106a-5p levels and further decreases the proliferation, and improves apoptosis rates.	(8)
	Blood samples of 103 OA and 100 normal cases	–	–	–	Increased H19 levels positively correlate with K-L grading and bone metabolism indexes β-CTX in OA patients.	(9)
NEAT1	30 OA and 30 normal tissues	–	miR‐193a‐3p, SOX5	–	By down-regulating miR‐193a‐3p levels, NEAT1 increases SOX5 and expands inflammation, apoptosis, and ECM degradation.	(15)
	30 OA and 30 normal tissues	–	miR-543, PLA2G4A	–	NEAT1 sponges miR-543 and increases PLA2G4A levels, therefore limiting the viability of chondrocytes. MMP levels proliferation rate were increased, and the apoptosis rate was decreased.	(16)
	15 OA and 10 control cases	–	miR-377-3p, ITGA6	–	NEAT1 knockdown up-regulates miR-377-3p, which significantly promotes chondrocyte proliferation and restrains inflammation, apoptosis, and ECM degradation.	(17)
DANCR	Synovial fluid-derived mesenchymal stem cells (SFMSCs) from 10 OA patients	–	miR-1275, Sox9, MMP-13	–	DANCR effectively decreases miR-1275 levels and further promotes Sox9 expression and SFMSCs proliferation and chondrogenesis.	(13)
	25 OA and 12 normal cases	–	miR‐19a	–	By restricting miR-19a in chondrocytes, DANCR escalates apoptosis and inflammation and lessens cell viability.	(11)
MALAT1	16 OA patients (8 obese and 8 normal-weight) and 6 normal controls	–	IL-6, CXCL8, TRIM6, IL7R, HIST1H1C, MAML3	ECM-receptor interaction, complement coagulation cascade	After cytokine stimulation, MALAT1 levels were increased. Due to the disrupted levels of its target genes, the proliferation rate of synovial fibroblasts was decreased.	(14)
	24 OA and 11 normal cases	–	miR-145, ADAMTS5	–	MALAT1 diminishes miR-145 levels and influences ADAMTS5 up-regulation in chondrocytes, limiting these cells’ viability and ECM degradation.	(24)
MFI2-AS1	46 OA and 28 normal cases	C28/I2	miR-130a-3p, TCF4	–	Increased MFI2-AS1 resulted in restricted viability and increased inflammation, ECM degradation, and apoptosis rate.	(25)
PART1	35 OA and 15 normal tissues	–	miR-373-3p, SOX4	–	PART1 increased SOX4 levels by decreasing miR-272-3p levels. Consequently, lower cell proliferation and extended apoptosis and ECM degradation rates were observed.	(26)
PVT1	40 OA patients: 20 with diabetes, and 20 without diabetes, 15 normal cases	–	miR-26b, CTGF	TGF-β	High glucose levels induce PVT1 expression, which further improves SMAD3, CTGF, TGF-β1, and MMP-13 expression and limits type II collagen levels.	(27)
	Blood samples 30 OA and 30 healthy controls	C28/I2	miR−93−5p, HMGB1	NF−κB	After LPS induction, PVT1 levels were elevated, and cell apoptosis and inflammation rates were extended.	(28)
TNFSF10	30 OA and 30 normal tissues	–	miR-376-3p, FGFR1	–	TNFSF10 up-regulates FGFR1 through modulating miR-376-3p expression. Moreover, this lncRNA improves cell proliferation, anti-apoptosis mechanisms, and inflammation in chondrocytes.	(29)
XIST	13 OA and 6 normal cases	THP-1	miR-376c-5p, OPN	–	XIST elevates the OPN levels, which enhances the macrophage M1 cytotoxicity. Subsequently, inflammation and apoptosis rates were increased.	(18)
	40 OA and 20 normal tissues	–	miR−1277−5p, ADAMTS5, MMP-13	–	XIST may promote ECM degradation by targeting miR-1277-5p and its downstream factors.	(19)
	–	SW1353, HEK293T	miR‐142‐5p, SGTB	–	XIST knockdown leads to miR‐142-5p up-regulation, increased proliferation, and ECM synthesis.	(20)
	15 OA and 7 normal tissues	–	TIMP-3	–	XIST binds to the TIMP-3 promoter and increases its methylation. Subsequently, in OA cases, increased collagen destruction was observed.	(21)
CHRF	–	ATDC5	miR-146a, IκBα, p65, JAK1, STAT3/IL‐6	NF-κB, JAK/STAT	CHRF increases apoptosis and inflammatory damages by inducing IL-6 expression.	(30)
CASC2	Blood samples from 71 OA and 55 healthy controls, synovial fluid samples from 21 OA and 15 healthy controls	CHON-001	IL-17	–	Increased CASC2 in chondrocytes results in elevated IL-17 levels and restricted proliferation.	(31)
FOXD2-AS1	35 OA and 35 normal cases	C28/I2	miR-27a-3p, TLR4/IL-1β, TNF-a	–	FOXD2-AS1 improves inflammation and ECM degradation in cells by down-regulating miR-27a-3p.	(32)
H19	–	C28/I2, HEK293T	miR-130a	–	After LPS induction, H19 levels, inflammatory factors, and apoptosis rate were increased.	(33)
TM1P3	35 OA and 10 normal cases	–	miR-22, SMAD1/5, MMP13, ALK1/IL‐1	TGF‐β	TIMP3, up-regulated by IL-1, diminishes miR-22 levels, and by affecting the TGF‐β pathway, the ECM degradation rate was increased.	(34)
THRIL	–	ATDC5	miR-125b, JAK1, STAT3	JAK/STAT, NF-κB	Up-regulation of THRIL intensified the LPS-induced inflammatory injury and apoptosis.	(35)
SNHG16	20 OA and 20 normal tissues	CHON−001	miR−373−3p, p21	–	SNHG16 decreases miR-373-3p and increases p21 levels, which further limits the viability and proliferation of cells. Collagen II and aggrecan levels were also reduced.	(36)
IGHC*γ*1	Blood samples of 88 cases and 36 healthy controls	THP-1	miR-6891-3p, TLR4	NF-κB	IGHCγ1 increased TLR4 expression through limiting miR-6891-3p, and improved macrophage proliferation, migration, and inflammatory responses.	(37)
CTBP1-AS2	62 OA and 62 healthy controls	–	miR-130a	–	CTBP1-AS2 methylates and deactivates miR-130a expression, which limits the proliferation rate.	(38)
LINC00511	–	ATDC5	miR-150-5p, SP1	–	LINC00511 down-regulation leads to expanded proliferation rate and ECM synthesis. By inducing a positive feedback loop, SP1 increases LINC00511 levels.	(39)
GAS5	Blood samples of 35 OA and 35 control cases	–	miR-137	–	By reducing miR-137 levels, GAS5 limits the proliferation rate of chondrocytes.	(40)
LINC00461	25 OA and 15 control cartilage tissues	–	miR-30a-5p	–	IL-6 and TNF-α promote LINC00461 expression, restrict miR-30a-5p levels, and further improved cell cycle progression, chondrocyte proliferation, inflammation, and ECM degradation.	(41)

While most of the studies which assessed the expression of NEAT1 or XIST in OA samples reported their up-regulation, few studies have demonstrated the opposite trend in their expressions. Lian et al. have reported down-regulation of XIST in OA chondrocytes. They have also shown protective effects of XIST in chondrocytes against IL-1β-induced damage through modulating the miR-653-5p/SIRT1 axis (42). Wang et al. have demonstrated down-regulation of NEAT1 in OA tissues, parallel with up-regulation of miR-181a. They have also shown that down-regulation of NEAT1 suppresses cell growth, elevates apoptosis, and increases the production of pro-inflammatory cytokines in OA chondrocytes (43). SNHG7 is another down-regulated lncRNA in OA. This lncRNA sponges miR-34a-5p and miR-214-5p (44, 45). Due to its sponging effects on miR-34a-5p, down-regulation of SNHG7 results in down-regulation of SYVN1, the direct target of this miRNA. Up-regulation of SNHG7 enhances cell proliferation and suppresses apoptosis and autophagy in OA cells (44). Also, SNHG7 can enhance cell viability and inhibit apoptosis and inflammatory responses in IL-1β-mediated chondrocytes through sponging miR-214-5p and up-regulating PPARGC1B expression. Thus, the protective effects of SNHG7 against OA are exerted through induction of the PPARγ pathway and combating the cytotoxic impact of miR-214-5p (45). The protective effects of other lncRNAs against OA are summarized in **Table 2**.

**Table 2:** Down-regulated lncRNAs in OA.

lncRNA	Clinical Samples	Assessed Cell Lines	Targets/Regulators	Signaling Pathways	Description	Reference
XIST	–	CHON-001, ATDC5	miR-653-5p, SIRT1	–	After IL-1β induction, XIST increased the viability of the cells while reducing the apoptosis and inflammation rates.	(42)
SNHG7	15 OA and 10 normal tissues	–	miR-34a-5p, SYVN1, Beclin1, LC3-II/I	–	SNHG7 decreases miR-34a-5p, which enhances proliferation and restricts the autophagy and apoptosis rate.	(44)
	30 OA and 12 normal tissues	–	miR-214-5p, PPARGC1B	–	SNHG7 down-regulates miR-214-5p and enhances cell viability.	(45)
MEG3	30 OA and 20 normal tissues	–	miR-361-5p, FOXO1	–	Down-regulated MEG3 effectively limits cell proliferation and curtails cell apoptosis and ECM degradation.	(46)
NEAT1	30 OA and 30 normal tissues	–	miR-181a, GPD1L	–	The knockdown of NEAT1 curbs cell growth while elevating the apoptotic rate and inflammatory cytokines.	
CAIF	60 OA and 60 normal tissues	CHON-001	miR-1246, IL-6	–	A diminished apoptosis rate was observed after miR-1246 reduced IL-6 due to CAIF reduction.	(47)
PART‐1	30 OA and 30 normal tissues	C20/A4	miR‐590‐3p, TGFBR2	TGF-β	PART‐1 down-regulation leads to decreased cell viability and promotes apoptosis rate.	(48)
NR024118	–	ATDC5	IL-1β, IL-6, IL-18	NF−κB, Nrf2	LPS lowers NR024118 expression and elevates the expression of IL-1β, IL-6, IL-18, and ROS. Furthermore, the inflammation, apoptosis, and oxidative stress rates were up-regulated.	(49)
MIR4435-2HG	Blood and tissue samples collected from 78 OA and 58 healthy controls	–	–	–	MIR4435-2HG up-regulation results in elevated proliferation rate and lower apoptosis rate.	(50)
SNHG1	–	IL-1β induced normal human articular chondrocytes-knee cells	miR-16-5p, ERK1/2, p38, p65	MAPK, NF-κB	SNHG1 up-regulation leads to diminished inflammation, metabolic dysfunction, and pro-inflammatory cytokines expression.	(51)
PACER	Plasma specimens from 73 OA and 66 healthy subjects	CHON-001	HOTAIR	–	PACER targets HOTAIR lncRNA and its overexpression results in a reduced apoptosis rate.	(52)
ANCR	Plasma specimens of 62 OA and 46 healthy cases	CHON‐001	TGF-β1	TGF-β	Up-regulating ANCR led to an enhanced proliferation rate by regulating the TGF-β signaling pathway.	(53)
DILC	Blood samples of 87 OA and 52 healthy subjects, synovial fluid from 22 OA, and 14 normal cases	CHON‐001	IL-6	–	DILC restricts IL-6 expression. However, it does not affect the proliferation and apoptosis rate of chondrocytes.	(54)
HULC	OA and normal cartilage tissue from 20 patients	ATDC5	miR-101	NF-κB, MAPK	HULC overexpression leads to down-regulated miR-101, which restricts cell inflammation.	(55)
LncRNA-ATB	–	ATDC5	miR-223	NF-κB, MAPK	After LPS induction, lncRNA-ATB levels were reduced, which resulted in miR-223 up-regulation and increased inflammation.	(56)
LINC00341	36 OA and 26 normal tissues	–	miR-141, YAF2	–	By down-regulating miR-141, LINC00341 increases YAF2 levels and restricts the apoptosis of chondrocytes.	(57)
SNHG5	25 OA and 25 normal controls	–	miR-10a-5p, H3F3B	–	SNHG5 hindered apoptosis and increased proliferation in IL-1β-stimulated chondrocytes by sponging miR-10a-5p.	(58)
SNHG9	60 OA and 60 normal subjects	–	miR-34a	–	SNHG9 increases miR-34a methylation and diminishes its expression, which further lowers the apoptosis rate.	(59)
SNHG15	20 OA and 10 normal cartilage tissues	–	miR-141-3, BCL2L13	–	SNHG15 increased BCL2L13 by down-regulating miR-141-3p, which led to a limitation in apoptosis and ECM degradation.	(60)
OIP5-AS1	35 OA patients and normal controls	CHON-001, ATDC5, HEK293	miR-29b-3p, PGRN	–	OIP5-AS1 overexpression results in improved proliferation and migration of chondrocytes and curtailed apoptosis rate and inflammatory responses.	(61)
CYTOR	52 OA and 52 normal subjects	402OA-05A, 402-05A	miR-10a-5p	–	miR-10a-5p is diminished after CYTOR up-regulation, which reduces the apoptosis rate.	(62)
NKILA	12 OA and 12 healthy controls	–	miR-145, SP1	NF-κB	NKILA increased and decreased the proliferation and apoptosis rates, respectively, by down-regulating miR-145 and up-regulating SP1.	(63)
HAND2-AS1	Blood samples of 67 OA and 34 normal controls	–	IL-6	–	The reduction in HAND2-AS1 level was correlated with aging and OA progression. However, its levels did not correlate with gender.	(64)
LINC00623	Chondrocyte isolation from normal and OA affected cartilage tissues	–	miR-101, HRAS	MAPK	LINC00623 increases HRAS levels by down-regulating miR-101, which leads to lower ECM degradation and apoptosis rates.	(65)
LUADT1	60 OA and 60 healthy cases	–	miR‐34a, SIRT1	–	LUADT1 down-regulation leads to miR-34a up-regulation and SIRT1 reduction. SIRT1, accordingly, increased the apoptosis rate of chondrocytes.	(66)

## miRNAs in Osteoarthritis

miRNAs are the utmost investigated small ncRNAs, representing an additional level of post-transcriptional controllers of gene expression that warrant the robustness of coordination in biological processes (67). These transcripts typically bind with the 3’ UTR of their target transcripts to either repress their translation or degrade them (68). In this section, we selected some miRNAs with clearer mechanistical information. Cheng et al. have reported up-regulation of miR-455-3p in the OA cartilages and IL-1β-exposed chondrocyte cells. This miRNA has been shown to partake in IL-1β-associated apoptosis and inflammatory responses. COL2A1 has been verified as a target of miR-455-3p designating the miR-455-3p/COL2A1 axis as a molecular mediator of OA (69). While confirming the role of miR-455-3p in OA’s chondrogenesis and development, Wen et al. have demonstrated down-regulation of this miRNA in the IL-1β model of OA. Over-expression of miR-455-3p has led to a significant decrease in PTEN and MMP13 while increases the COL2A1 expression levels. Moreover, based on their observations, miR-455-3p can decrease chondrocytes’ apoptotic rate by affecting PTEN expression (70). Despite using similar OA models, these studies have reported conflicting results regarding the role of miR-455-3p in the development of OA. Wand et al. have demonstrated up-regulation of miR-1236 in OA-affected cartilages compared to normal cartilages. Such up-regulation has inhibited chondrocyte proliferation and induced apoptosis in these cells through targeting PIK3R3 (71). miR-411 is another up-regulated miRNA in OA models. This miRNA directly affects the expression of HIF-1α. LC3, ULK-1, P62, and Beclin-1 have been among genes whose expressions have been affected by miR-411. miR-411 has been shown to enhance chondrocyte autophagy through modulating HIF-1α (72). miR-203 is another miRNA whose expression has been promoted by IL-1β stimulation. This miRNA enhances cellular inflammatory responses and cell damage and reduced aggrecan and Col2A1 levels. miR-203 binds with ERα and exerts its effects in OA development through this axis (73). miR-140 and miR-199 are two down-regulated miRNAs in the synovial tissues of OA patients compared with healthy controls. Expressions of these miRNAs have been shown to decrease during the course of OA. Moreover, their expressions have been inversely correlated with the severity of OA (74). The course of OA has been found to be alleviated by exosomal miR-9-5p produced by mesenchymal stem cells originated from bone marrow. This miRNA has been shown to decrease syndecan-1 levels and diminish pro-inflammatory cytokines as well as CRP (75). **Tables 3** and **4** show the up-regulated and down-regulated miRNAs in OA, respectively.

**Table 3:** Up-regulated miRNAs in OA.

miRNA	Clinical Samples	Assessed Cell Lines	Targets/Regulators	Signaling Pathways	Description	Reference
miR-455-3p	30 OA and 30 control cases	CHON-001	COL2A1	–	miR-455-3p increases the IL-1β-induced apoptosis and inflammation rates by targeting the COL2A1 directly.	(69)
miR-1236	9 OA and 9 control cartilage tissues	–	PIK3R3	–	Up-regulated miR-1236 restricts the proliferation rate in chondrocytes.	(71)
miR-411	–	C28/I2	HIF-1α, LC3, ULK-1, P62, Beclin-1	–	miR-411 down-regulates HIF-1α and enhances the autophagy rate of chondrocytes.	(72)
miR-203	Cartilage and blood samples of 34 OA and 20 normal cases	–	ERα, Col2A1	–	miR-203 was up-regulated after IL-1β induction, which led to chondrocyte injury, inflammation, and diminished aggrecan and Col2A1 levels.	(73)
miR-103	7 OA and 23 control tissues	–	SPHK1	PI3K/AKT	miR-103 overexpression results in diminished SPHK1 and cell proliferation, while the apoptosis rate elevates.	(76)
miR-27a	20 OA and 10 normal tissues	SW1353	PI3K	PI3K/AKT	miR-27a down-regulation regulates the PI3K/AKT signaling pathway and lowers the apoptosis rate.	(77)

**Table 4:** Down-regulated miRNAs in OA.

miRNA	Clinical Samples	Assessed Cell Lines	Targets/Regulators	Signaling Pathways	Description	Reference
miR-455-3p	5 OA samples, 5 healthy chondrocyte donors, 4 bone marrow stem cells donors	–	PTEN	PI3K/AKT	By regulating the PI3K/AKT pathway, miR-455-3p diminishes the apoptosis rate.	(70)
miR-140-5p	12 OA and 12 normal cases	–	HMGB1	PI3K/AKT	miR-140-5p suppresses HMGB1 expression and prohibits MMP expression, inflammation, and apoptosis.	(78)
miR-149-5p	56 OA and 32 healthy controls	–	AGT	JAK/STAT	miR-149-5p up-regulation results in reduced AGT, which blocks the RAS system and hampers MMP-13 and nitrite in chondrocytes.	(79)
miR-140	110 OA and 60 healthy individuals	–	–	–	miR-140 and miR-199 levels inversely correlate with OA severity, MMP-3 expression, and IL-1β mRNA levels.	(74)
miR-199						
miR-93-5p	60 OA and 60 healthy controls	402OA-05A	CASC2	–	Up-regulated miR-93-5p dwindles the apoptosis of chondrocytes induced by LPS through CASC2.	(80)
miR-26a-5p	21 OA and 15 normal tissues	–	PTGS2	–	miR-26a-5p curtails PTGS2 levels and the damage on synovial fibroblasts.	(81)
miR-33b-3p	38 OA and 38 healthy tissues	CHON-001	IRAK3	–	miR-33b-3p overexpression hinders IRAK3 and leads to lower inflammatory cytokine expression and apoptosis rates.	(82)
miR-335-5p	6 OA and 6 normal controls	–	GAG, Beclin-1, ATG5, ATG7	–	miRNA-335-5p increases the viability and autophagy-related factors expression by up-regulating GAG. Furthermore, apoptosis and inflammation rates were hindered by this miRNA.	(83)
miR-320c	6 OA and 6 normal cartilage tissues	–	β-catenin	Wnt	miR-320-3p promotes cartilage production and chondrogenesis by targeting the Wnt signaling pathway.	(84)

## CircRNAs in Osteoarthritis

These ncRNAs have a circular conformation shaped by routine spliceosome-mediated or lariat kind of splicing (85). Exonic circRNAs, circular intronic RNAs, exonic-intronic circRNAs, and tRNA intronic circRNAs constitute the main classes of circRNAs (85). Circ_0136474 is a member of this group which can inhibit cell proliferation by enhancing MMP-13 expression and decreasing miR-127-5p levels in OA (86). Hsa_circ_0005105 is another up-regulated circRNA in IL-1β-induced chondrocytes. Hsa_circ_0005105 has been shown to suppress transcriptional activity of miR-26a, thus up-regulating expression of NAMPT, the direct target of this miRNA. Moreover, hsa_circ_0005105 can decrease the levels of type II collagen and aggrecan, enhance MMP-13 and ADAMTS-4 levels, and increase the production of PGE2, IL-6, and IL-8 (87). CircHIPK3 is another circRNA that regulates the apoptosis rate of chondrocytes through the miR-124/SOX8 axis (88). On the other hand, circRNA-UBE2G1 mainly regulates OA development through influencing inflammatory responses. This circRNA targets miR-373 and increases IL-1β, IL-6, and TNF-α levels in LPS-treated cells (89). **Tables 5** and **6** show the list of up-regulated and down-regulated circRNAs in OA, respectively.

**Table 5:** Up-regulated circRNAs in OA.

circRNA	Clinical Samples	Assessed Cell Lines	Targets/Regulators	Signaling Pathways	Description	Reference
Circ_0136474	7 OA and 7 normal cartilage samples	–	miR‐127‐5p, MMP‐13	–	Circ_0136474 suppresses miR-127 and elevates MMP-13 expression. Hence, the apoptosis rate reduces through the diminished IL‐1β, TNF‐α, IL‐17, and elevated type II Collagen.	(90)
hsa_circ_0005105	–	4651-SC	miR-26a, NAMPT	–	By targeting miR-26a, this circRNA elevates the NAMPT expression. Accordingly, it escalated the production of inflammatory factors occurs.	(87)
CircHIPK3	36 OA and 36 control cases	–	miR-124, SOX8	–	CircHIPK3 increased the apoptosis rate of chondrocytes by elevating the SOX8 expression through depleting miR-124.	(88)
circRNA-UBE2G1	53 OA and 13 healthy tissues	C28/I2	miR-373, HIF-1a	–	This circRNA hampers the expression of inflammatory cytokines, such as IL-1β, IL-6, and TNF-α in LPS-treated cells by targeting miR-373 expression.	(89)
CircPSM3	35 OA and 35 control cases	–	miR-296-5p	–	CircPSM3 reduces the proliferation and differentiation of chondrocytes through down-regulating miR-296-5p.	(91)
ciRS-7	Cartilage samples from OA and trauma patients	C28/I2	miR-7, IL-17, Beclin1, LC3-II/I, p62	PI3K/AKT	Up-regulated ciRS-7 leads to down-regulated miR-7 expression, which extends the IL-1β-induced cartilage degradation, and lessens the autophagy rate.	(92)

**Table 6:** Down-regulated circRNAs in OA.

circRNA	Clinical Samples	Assessed Cell Lines	Targets/Regulators	Description	Reference
CircRNA-9119	20 OA and 10 normal cases	SW1353	miR-26a, PTEN	Up-regulated circRNA-9119 diminishes miR-26a and improves the viability of chondrocytes.	(93)
CircSERPINE2	30 OA and 30 normal cases	–	miR-1271-5p, COL2A1, aggrecan, MMP3, MMP13	Down-regulated CircSERPINE2 stimulates apoptosis and ECM destruction by targeting miR-1271-5p and its downstream factors.	(94)
circANKRD36	36 OA and 9 normal tissues	–	miR‐599, Casz1	CircANKRD36 significantly reduces the apoptosis and inflammation rates of chondrocytes.	(95)

## Animal Studies

In addition to cell line studies and expression assays in human samples, the expression and function of ncRNAs have been appraised in OA animal models. Commonly, the observed dysregulation of ncRNAs in the animal models is consistent with findings in human-derived OA tissues and *in vitro* studies. Moreover, deregulation of these transcripts has similar outcomes in the animals and in *in vitro* studies. In fact, animal studies have provided strong evidence for functionality of these transcripts in the pathogenesis of OA. As an example, HOTAIR has been shown to be elevated in articular cartilage samples of OA mice in association with down-regulation of miR-20b and up-regulation of PTEN. HOTAIR knockdown has ameliorated cartilage tissue injury in animal models and enhanced collagen II and aggrecan levels in this tissue while decreasing MMP-13 and ADAMTS-5 levels (96). LOC101928134 and LINC00662 are two other lncRNAs whose functions in OA development have been investigated in animal models (97, 98). While LOC101928134 increases apoptosis and cartilage damage through activation of the JAK/STAT signaling pathway (97), LINC00662 reduces apoptosis and inflammatory factors such as IL-6 and IL-8 (98). miR-34a, miR−363−3p, miR‐101a‐3p, circRNA.33186, and circRNA_Atp9b are other ncRNAs whose roles in the development of OA have been appraised in animal models (**Table 7**).

**Table 7:** Summary of studies that reported the role of ncRNAs in animal models of OA.

ncRNA	Expression Pattern	Animal model	Targets/regulators	Signaling Pathway	Description	Reference
HOTAIR	Up	Male adult C57BL/6 mice, OA model was induced by medial collateral ligament transection and DMM.	miR-20b, PTEN	PTEN	HOTAIR expression results in diminished collagen II and aggrecan and improved MMP-13 and ADAMTS-5 expression. This lncRNA further declined the proliferation and heightened ECM destruction.	(96)
LOC101928134	Up	Sprague-Dawley rats, OA was induced by anterior cruciate ligament Transection.	IFNA1	JAK/STAT	This lncRNA elevates IFNA1 and activates JAK/STAT signaling pathway. Consequently, apoptosis and cartilage damage rates were increased.	(97)
LINC00662	Down	Male Sprague‐Dawley rats, OA was induced by medial capsular incision.	miR‐15b‐5p, GPR120	–	After LINC00662 reduction, miR-15b-5p is increased, which results in reduced GRP120 levels. Consequently, apoptosis and inflammatory factors such as IL-6 and IL-8 were elevated.	(98)
miR-34a	Up	male Sprague Dawley rats were subjected to anterior cruciate ligament transection.	–	PI3K/AKT	miR-34a decreases the proteins involved in PI3k/AKT pathway and increases the apoptosis rate of chondrocytes.	(99)
miR−363−3p	Up	Male Wistar rats were subjected to medial meniscectomy tear surgery.	NRF1	p53	miR−363−3p elevates the apoptosis rate by enhancing IL−1β, IL−6, and TNF−α expression.	(100)
miR‐101a‐3p	Down	Sprague Dawley rats were injected with Complete Freund’s Adjuvant emulsion into the upper TMJ cavities.	UBE2D1, FZD4	Wnt	miR‐101a‐3p significantly improves the apoptosis of chondrocytes by regulating the Wnt signaling pathway.	(101)
circRNA.33186	Up	Adult male C57BL/6 mice were subjected to DMM surgery.	miR-127-5p, MMP-13, Col2a1	–	circRNA.33186 down-regulates miR-127 and up-regulates MMP-13 expression, which leads to diminished cell proliferation rate.	(102)
circRNA_Atp9b	Up	Mouse articular chondrocytes obtained from the knee joints	miR-138-5p, MMP13, IL-6, COX-2	–	CircRNA_Atp9b down-regulation increases collagen type II and inhibits MMP13, COX-2, and IL-6 expression, resulting in ECM degradation and inflammation.	(103)

## Action of ncRNAs Upon Key Pathways in Osteoarthritis

NcRNA can affect pathoetiology of OA through different routes such as JAK/STAT, NF-κB, PI3K/AKT and Wnt/β-catenin signaling pathways as well as autophagy. It is worth mentioning that a single ncRNA might affect pathogenesis of OA through modulation of different pathways. For instance, HOTAIR has been found to affect both PI3K/AKT signaling and autophagy, thus it has a pleiotropic role in OA. In fact, these effects might be complementary to each other to worsen disease progression. Similarly, the same signaling pathway can be affected by many different ncRNAs at different points. One might deduce that these ncRNAs act in a timely-concerted manner, yet no study has assessed the effects of these ncRNAs at different regulatory points of signaling pathways or during the course of OA. Thus, there is no proof for this hypothesis based on the currently available literature.

**Figure 1:**
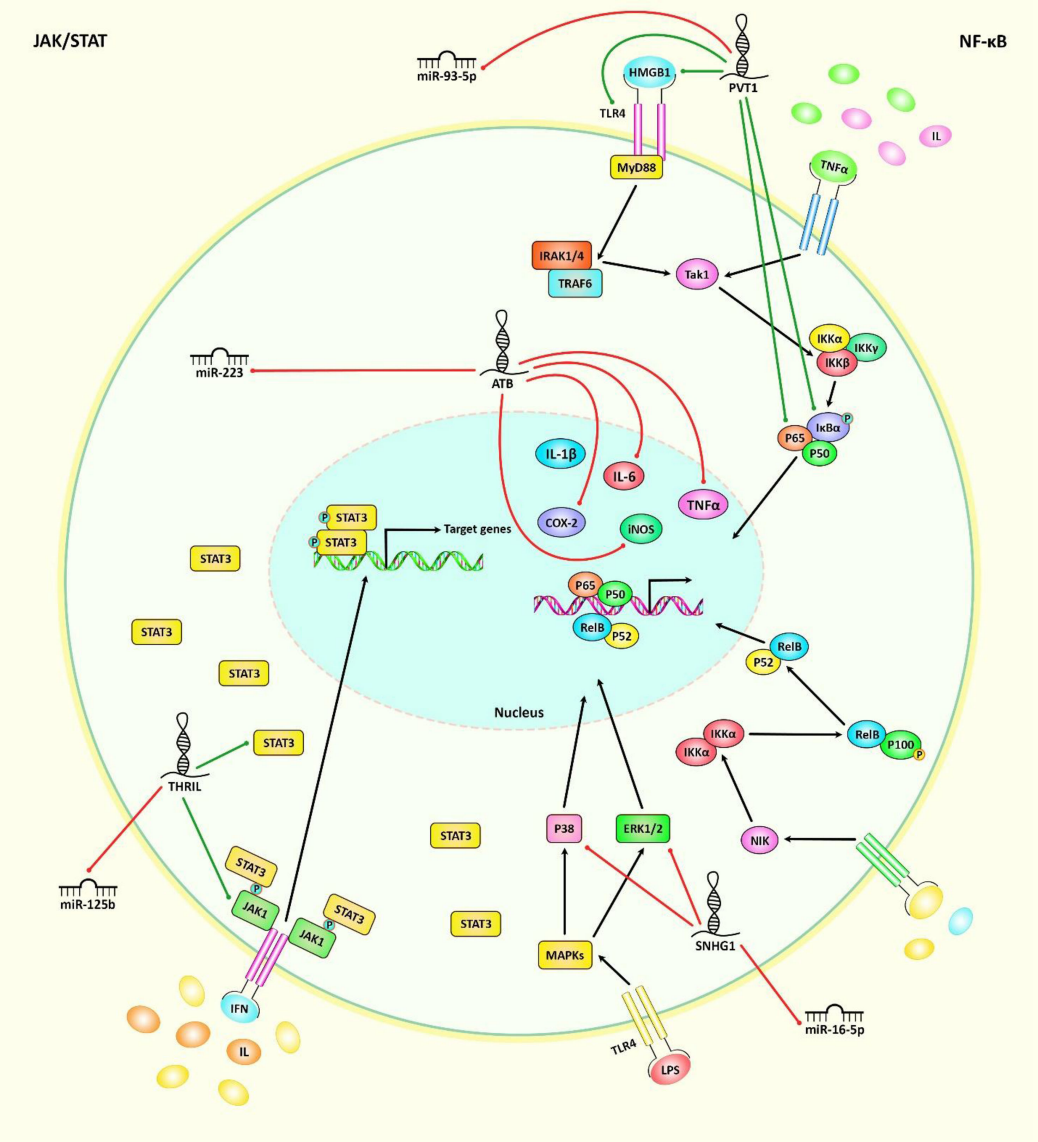
A schematic illustration of the role of various noncoding-RNAs in modulating the JAK/STAT and NF-κB signaling pathways in osteoarthritis. Mounting studies have revealed that multiple ncRNAs (lncRNAs, circRNAs, and miRNAs) have important roles in osteoarthritis through regulating the JAK/STAT and NF-κB cascades. As an illustration, lncRNA PVT1 could play an effective role in upregulating TLR4/NF-κB signaling cascade *via* modulating miR-93-5p/HMGB1 axis in osteoarthritis patients, therefore inducing osteoarthritis development (28). In addition, lncRNA-ATB overexpression could have a crucial part in downregulating the expression levels of iNOS, COX-2, IL-6 and TNF-α proteins. These lncRNA could reduce miR-223 expression through suppressing MyD88/NF-κB and p38MAPK cascades, and thereby alleviating lipopolysaccharide-induced inflammatory injury in osteoarthritis patients (56). Another study has confirmed that lncRNA SNHG1 through downregulating the expression levels of IL-6, TNF-α, iNOS, COX-2, ERK1/2, P38, and P65 as well as suppressing miR-16-5p-mediated p38MAPK and NF-κB signaling cascades could have an effective role in alleviating IL-1β-induced osteoarthritis (51). Green arrows indicate the upregulation of target genes modulated *via* ncRNAs (lncRNAs, circRNAs, and miRNAs), red arrows depict inhibition regulated by these ncRNAs. All the information regarding the role of up-regulated or down-regulated ncRNAs in modulating osteoarthritis can be seen in **Tables 1**–**7**.

Activation of JAK/STAT signaling pathway acts as a common connection linking pro-inflammatory cytokines to inflammation in the context of OA (104). In addition, expression of the NF-κB family of transcription factors can be induced by pro-inflammatory cytokines and chemokines as well as degradation products of extracellular matrix. Activation of NF-κB molecules can increase expression of several genes which increase damage to the articular joint, thus participating in the pathogenesis of osteoarthritis (105). A number of ncRNAs can affect pathogenesis of OA *via* modulation of these pathways. **Figure 1** illustrates the role of various ncRNAs in regulating the JAK/STAT and NF-κB signaling pathways in OA.

**Figure 2:**
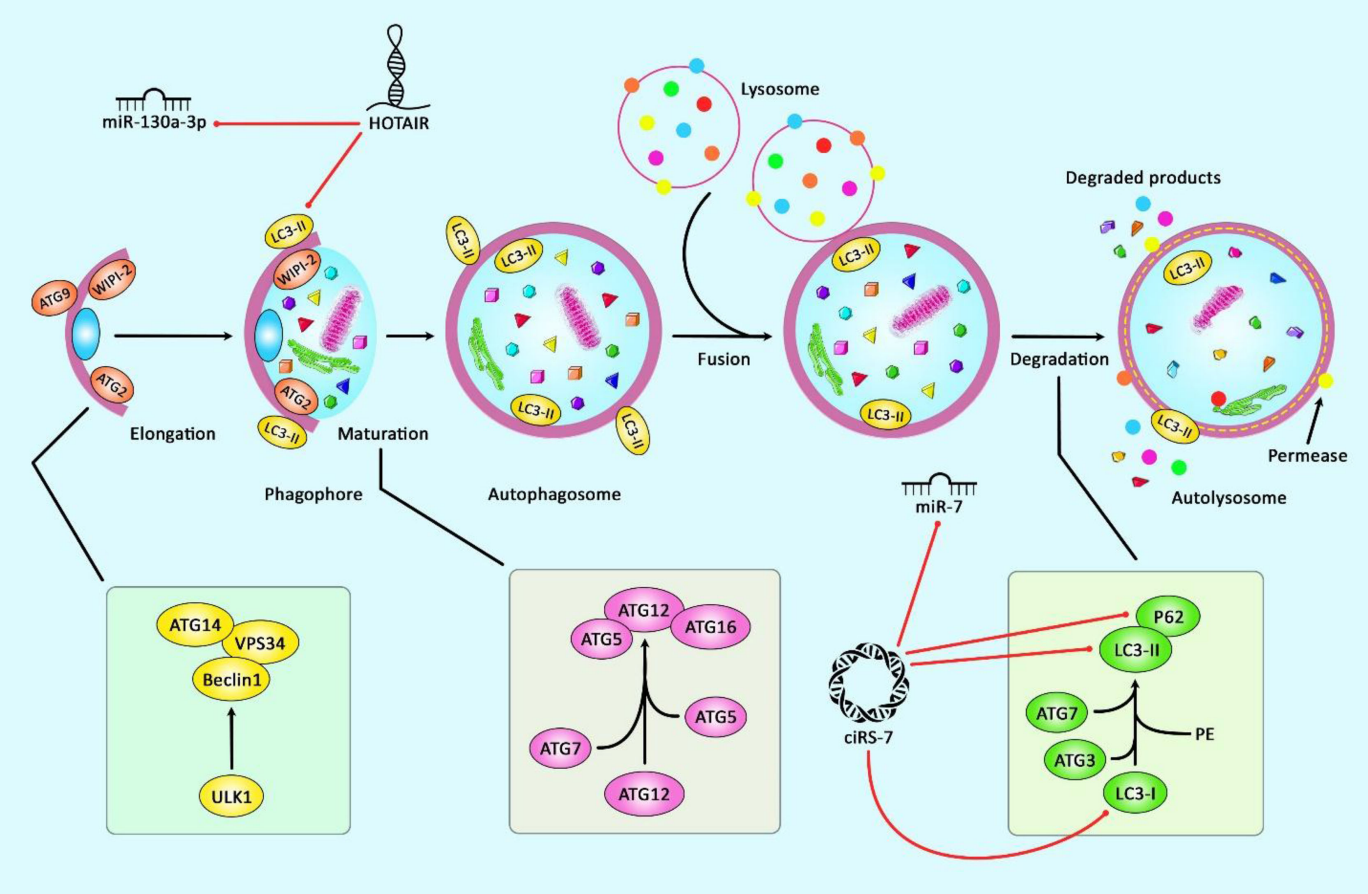
A schematic representation of the role of several ncRNAs in regulating the autophagy cascade in osteoarthritis. Growing evidence confirm that several ncRNAs could regulate the autophagy pathway in osteoarthritis. A recent study have authenticated that lncRNA HOTAIR through downregulating the expression levels of miR‐130a‐3p, P62, LC3 I, LC3 II could play a significant role in suppressing chondrocyte autophagy in knee osteoarthritis (10). Moreover, according to another research, ciRS-7 through sponging miR-7 and reducing the expression levels of LC3, p62, and Beclin1 could reduce cartilage degradation and attenuate autophagy cascade in osteoarthritis *via* modulating PI3K/AKT/mTOR pathway (92). Red arrows indicate downregulation of target genes by ncRNAs.

The abnormal alterations in the course of osteoarthritis mostly are linked with dysfunction of chondrocytes and autophagy, an intracellular mechanism of degradation that preserves the stable condition of cellular metabolism. This process is also regarded as a mechanism for restoring activity of injured chondrocytes. Thus, it has a role in alleviation of OA (106). **Figure 2** represents the role of several ncRNAs in OA through regulating the autophagy pathway.

**Figure 3:**
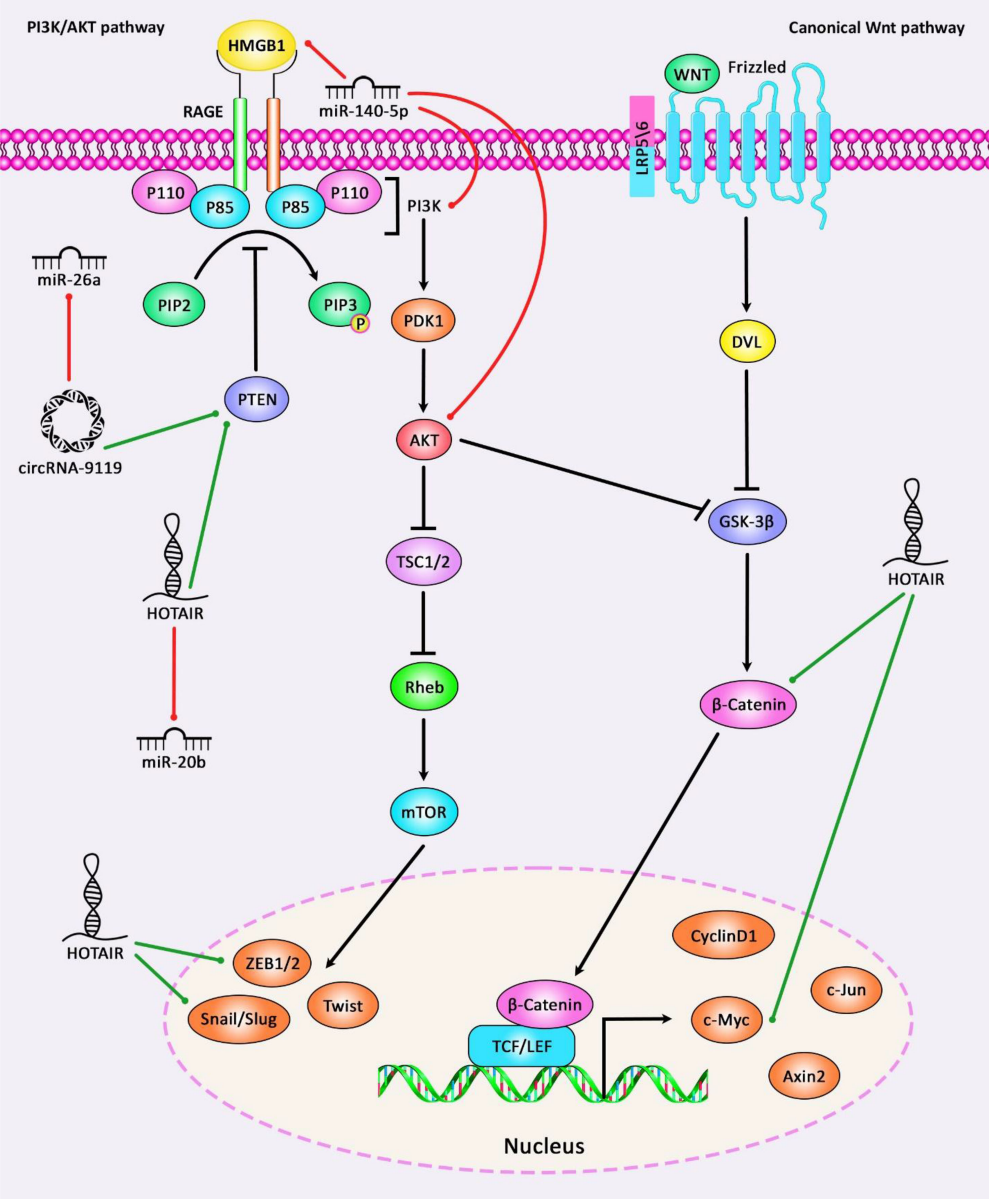
A schematic diagram of the role of some ncRNAs in modulating the PI3K/AKT and Wnt/β-catenin signaling pathways in osteoarthritis. Current research has demonstrated that circRNA-9119 *via* sponging miR-26a could have a significant part in promoting the expression level of PTEN. These circRNA could suppress IL-1β-induced chondrocyte apoptosis, and possibly triggering Osteoarthritis progression (93). Moreover, another study has denoted that lncRNA HOTAIR could enhance the activation of Wnt/β-catenin signaling cascade *via* downregulating WIF-1 expression in osteoarthritic chondrocytes by promoting the expression levels of c-Myc, ZEB1, and Snail as downstream target genes of Wnt/β-catenin signaling, thereby elevating catabolic gene expression and increasing cartilage degradation (7). Green arrows indicate upregulation of target genes *via* ncRNAs (lncRNAs, circRNAs, and miRNAs), red arrows depict inhibition by these ncRNAs. All the information regarding the role of these ncRNAs in modulating the PI3K/AKT and Wnt/β-catenin cascades in osteoarthritis can be seen in **Tables 1**–**7**.

Wnt signaling has an important role in osteoarthritis pathogenesis and is regarded as a potential target for treatment of this disorder. Wnt pathway contains 19 Wnt genes and several receptors regulating canonical and non-canonical pathways, the latter being dependent on β-catenin. Wnt signaling has important roles in the regulation of proliferation and differentiation of cells, as well as their polarization (107). PI3K/AKT/mTOR signaling pathway is another pathway which has important roles in the normal metabolic pathways in the joints and participates in the development of OA through induction of cartilage degradation, impairment of function of subchondral bones, and induction of inflammatory responses in the synovial tissues (108). **Figure 3** illustrates the role of different ncRNAs in OA through modulating the PI3K/AKT and Wnt/β-catenin signaling pathways.

## Association Between ncRNAs Polymorphisms and OA

H19, MEG3, and PRNCR1 are three lncRNAs whose polymorphisms have been associated with OA’s risk. For instance, the A allele of the rs217727 within H19 can enhance the risk of OA. However, the rs3741219 within this lncRNA has not affected the risk. Notably, the rs217727 polymorphism has been associated with the levels of H19, hsa-miR-4804-5p, hsa-miR-8071, hsa-miR-8072, and hsa-miR-3960 in the circulation. Besides, the A allele of the rs7158663 within MEG3 can increase the risk of OA. rs7158663 has been associated with the plasma levels of its host genes, i.e. hsa-miR-4307 and hsa-miR-1265 (109). Finally, the G allele of rs1456315 within PRNCR1 can increase the risk of OA (110).

An integrative analysis of lncRNAs association with OA has shown that RegulomeDB scores of three SNPS within H19, MEG3 and HOTTIP have been 2b (111). Based on the ChIP-seq data, these SNPs can bind with EZH2, E2F6, REST and IKZF1 proteins (http://regulome.stanford.edu/) (111). Previous studies have shown the involvement of these proteins in the pathogenesis of OA or regulation of cellular functions. For instance, suppression of EZH2 can ameliorate development of OA *via* modulation of Wnt/β-catenin pathway (112). E2F6 is involved in the regulation of cell cycle (113). REST encodes a transcriptional repressor which suppresses neuronal genes in non-neuronal tissues (114). IKZF1 is involved in the chromatin remodeling (115). Its role in the regulation of inflammation implies its involvement in the pathogenesis of OA (116).

Although the mechanisms behind involvement of these SNPs in the pathogenesis of OA have not been completely understood, it is possible that these SNPs affect interaction with other target RNAs. Moreover, they can influence expression levels of ncRNAs, thus affecting their regulatory effects.

Identification of risk variants for development of OA can help in development of novel OA therapeutic approaches such as gene editing or gene replacement therapies for OA. **Table 8** summarizes these studies.

**Table 8:** Association between ncRNAs polymorphisms and OA.

lncRNA	Number of Clinical Samples	SNP ID	Nucleotide change	Description	References
H19	230 Han Chinese OA patients and 230 healthy subjects, matched by age and gender	rs217727	G>A	“A” allele of the rs217727 of H19 increases the risk of OA.	(109)
MEG3		rs7158663	A>G	Having the “A” allele of the rs7158663 of MEG3 increases the risk of OA by 1.32.	
PRNCR1	316 OA and 306 healthy cases	rs1456315	A>G	Mutant G allele of PRNCR1 rs1456315 increases the risk of OA.	(110)
H19	278 Knee OA patients and 289 controls	rs2067051	T>C	T allele of rs2067051 was associated with lower susceptibility to knee OA.	(111)
MEG3		rs4378559	C>T	T allele of rs4378559 was associated with higher susceptibility to knee OA.	
HOTTIP		rs202384		C allele of rs2023843 showed boundary positive in additive genetic model.	

## Diagnostic Roles of ncRNAs in OA

Recent investigations have tested the appropriateness of ncRNAs in diagnostic purposes in OA. Circulating ncRNAs (in blood/plasma/serum) are definitely one of the most interesting biomarkers for OA due to the easy accessibility of sample. Although synovial fluid samples have also been applicable for this purpose, blood/plasma/serum samples are superior since they are obtained through less invasive methods. For instance, expression levels of H19 in the blood samples could distinguish OA cases from normal subjects with AUC, critical, sensitivity, and specificity values of 0.89, 1.87, 96%, and 85.7%, respectively (9). In a study with limited numbers of cases and controls, GAS5 has been shown to predict the presence of OA with an accuracy of 0.86 (40). The highest diagnostic power among lncRNAs has been achieved by MIR4435-2HG (AUC=0.96) (50). Hsa_circ_0032131 is the only circRNA whose appropriateness for diagnostic strategies in OA has been appraised (117). **Table 9** gives an overview of the diagnostic impact of ncRNAs in OA, based on the studies that assessed expression of these transcripts in the circulation of patients. These ncRNAs are merely exemplificative of the work being published in the field.

**Table 9:** Diagnostic role of ncRNAs in OA.

lncRNA and Clinical Cases	AUC	Sensitivity	Specificity	Reference
H19 expression in blood samples of 103 OA and 100 control subjects	0.891	96.00	85.73	(9)
GAS5 expression in the blood samples of 2 groups, OA and control cases, each with 35 cases	0.860	–	–	(40)
MIR4435-2HG expression measured in blood samples of 78 OA and 58 healthy cases	0.96	–	–	(50)
PACER expression in plasma samples of 73 OA and 66 healthy controls	0.95	–	–	(52)
HOTAIR expression in plasma samples of 73 OA and 66 healthy controls	0.90			
ANCR expression in the plasma specimens of 62 OA and 46 healthy cases	0.8845	–	–	(53)
DILC expression in the plasma of 87 OA and 52 healthy subjects	0.9321	–	–	(54)
hsa_circ_0032131 expression in blood samples of 25 OA and 25 healthy cases	0.8062	0.90	0.65	(117)
Plasma levels of miR-200c-3p in 150 OA cases and 150 controls	0.755	–	–	(118)
Plasma levels of miR-100-3p in 150 OA cases and 150 controls	0.845	–	–	
Plasma levels of miR-1826 in 150 OA cases and 150 controls	0.749	–	–	

## Discussion

OA is a multifactorial disorder in which several classes of ncRNAs, including lncRNAs, circRNAs, and miRNAs participate. Notably, the two former classes of ncRNAs mainly exert their effects in this process through acting as molecular sponges for miRNAs. These ncRNAs collaborate to influence chondrocyte proliferation and apoptosis, inflammatory responses, and degradation of ECM. Studies that investigated ncRNAs’ role in OA can be classified according to their design to *in vitro* studies, expression assays in clinical samples, and functional studies in animal models. The latter type of studies has provided essential concepts about the role of ncRNAs in this process, as it could assess these transcripts’ functional roles in a natural context. Meanwhile, clinical studies, particularly those assessing expression levels of ncRNAs in the peripheral blood, have the advantage of discovering appropriate markers for the diagnosis of OA and prediction of its course.

NcRNAs can be involved in the fine tuning of the RUNX2 expression and through this rout, they can affect pathogenesis of OA (119, 120). NF-κB, Wnt/β-catenin, TGF-β and JAK/STAT pathways are the most critical pathways through which ncRNAs exert their effects in the pathogenesis of OA. Based on the functional relevance of these pathways with inflammatory responses, one can conclude that this process has a prominent role in the development of OA. A PPAR-α agonist has been found to inhibit LPS-associated inflammatory responses in synovial fibroblasts through modulation of NF-κB signaling (121). Therefore, ncRNAs associated with these pathways might also represent therapeutic targets for OA.

The cartilage tissue has been mainly studied for the assessment of the ncRNAs’ expression. However, limited numbers of studies have investigated the expression of these transcripts in patients’ synovial membrane or peripheral blood, evaluating their continuation as non-invasive markers for the detection of OA. These studies have reported diagnostic power values ranging from 0.80 to 0.96. Nevertheless, most of these studies have been conducted in limited numbers of cases and controls, precluding the generalization of their results.

The data presented above shows involvement of several ncRNAs in the pathoetiology of OA. This information can be used for design of novel therapeutic options for this disorder. Moreover, it can be used to find genetically susceptible people to OA. However, further assessment of applicability of ncRNAs-targeting treatment modalities in animal models is a prerequisite for translation of this filed of basic science into clinical application.

## Conclusion

Despite valuable research, this field lacks a comprehensive assessment of different classes of ncRNAs in OA samples. Such study would increase our understanding of the functional relationship between circRNAs, lncRNAs, and miRNAs, thus expanding our knowledge about the pathobiology of OA.

Another gap in this field is the scarcity of assessment of the impact of functional polymorphisms within ncRNAs in conferring OA risk and modulating the disease course. Identification of genomic variants that affect the risk of OA would help in the modification of lifestyle in order to attenuate the course of the disorder.

## Author Contributions

SG-F wrote the draft and revised it. MT designed and supervised the study. CP and MM revised the draft. AT collected the data and designed the figures and tables. All the authors read and approved the submitted version.

## Conflict of Interest

The authors declare that the research was conducted in the absence of any commercial or financial relationships that could be construed as a potential conflict of interest.

## Publisher’s Note

All claims expressed in this article are solely those of the authors and do not necessarily represent those of their affiliated organizations, or those of the publisher, the editors and the reviewers. Any product that may be evaluated in this article, or claim that may be made by its manufacturer, is not guaranteed or endorsed by the publisher.
